# 

**Published:** 1908-04-11

**Authors:** 


Telegraphic Address: Ospedale, London.

THE MODERN MEDICAL JOURNAL.

Telephone Number: 2734 Gerrard.

NEW SERIES. ~~No. 56, Vol. III.~~ No. 1128. Vol. XLIV. — SATURDAY, APRIL 11TH, 1908. — PRICE THREEPENCE.

